# Low-Illumination Image Enhancement in the Space Environment Based on the DC-WGAN Algorithm

**DOI:** 10.3390/s21010286

**Published:** 2021-01-04

**Authors:** Minglu Zhang, Yan Zhang, Zhihong Jiang, Xiaoling Lv, Ce Guo

**Affiliations:** 1 School of Mechanical Engineering, Hebei University of Technology, Tianjin 300130, China; zhangml@hebut.edu.cn (M.Z.); 201811201017@stu.hebut.edu.cn (Y.Z.); 201921202077@stu.hebut.edu.cn (C.G.); 2 The Intelligent Robotics Institute, School of Mechatronic Engineering, Beijing Institute of Technology, Beijing 100811, China; w15022139757@163.com

**Keywords:** low-illumination image, CIELAB, DC-WGAN, image enhancement

## Abstract

Owing to insufficient illumination of the space station, the image information collected by the intelligent robot will be degraded, and it will not be able to accurately identify the tools required for the robot’s on-orbit maintenance. This situation increases the difficulty of the robot’s maintenance in a low-illumination environment. We proposes a novel enhancement method for images under low-illumination, namely, a deep learning algorithm based on the combination of deep convolutional and Wasserstein generative adversarial networks (DC-WGAN) in CIELAB color space. The original low-illuminance image is converted from the RGB space to the CIELAB color space which is relatively close to human vision, to accurately estimate the illumination image, and effectively reduce the effect of uneven illumination. DC-WGAN is applied to enhance the brightness component by increasing the width of the generation network to obtain more image features. Subsequently, the LAB is converted into RGB space to obtain the final enhanced image. The feasibility of the algorithm is verified by experiments on low-illuminance image under general, special, and actual conditions and comparing the experimental results with four commonly used algorithms. This study lays a technical foundation for robot target recognition and on-orbit maintenance in a space environment.

## 1. Introduction

Space particle radiation poses a serious threat to the health of astronauts. Intelligent robots are unrestricted by human physiological conditions. Thus, the use of these robots to assist astronauts in space utilization and detection in harsh environments is an inevitable choice in the development of the space station automation technology. This trend is also an important development planning for international and Chinese space stations [1,2,3,4]. The space station orbits the Earth in approximately 90 min. The air is thin, and the side facing the Sun is unobstructed and exposed to direct light. Meanwhile, the other side faces endless darkness, and the illuminance sharply drops. Furthermore, the unique space environment (e.g., excessive brightness or darkness) and the mirror-like coating of the space device can cause multiple reflections, particle radiation, and other convoluted interferences [5], which might lead to severe interference to the operation target characteristics. In addition, the accuracy of intelligent robot target recognition poses a challenge. Pre-processing each frame of collected images, especially low-illumination images, is conducive to the accurate identification of the goal. In addition to the four-color cameras on the head of the Robotnaut2 robot astronaut developed by the National Aeronautics and Space Administration (NASA), an infrared time-of-flight camera is also attached to the robot to provide the depth of field information. Solving optical image interference in the complex space environment is challenging [6]. In 2016, NASA adopted the visual image processing algorithm of Robonaut 2 to the complex space environment of the world to replace the algorithm used by the current visual sensor and help the Robonaut 2 robot astronauts in reliably identifying handles, tools, and other moving targets [7]. The Beijing Institute of Technology conducted simulation experiments on the ground and practical applications in the Tiangong-2 laboratory under stable light conditions. The results show that the low-illumination image enhancement can accurately identify the target and improve the accuracy of the robot to grasp the blob [8]. However, the image processing algorithm for complex interference caused by poor lighting in space is not yet developed. Thus, improving the image quality obtained by space intelligent robots and increasing the accuracy for the subsequent target recognition are urgent problems that must be solved.

This study investigates the negative effects caused by the tendency of low-illumination environments to conceal target feature information. The commonly used processing methods are classified into three categories. The first category is histogram equalization (HE), which balances the histogram of the whole image. Gamma correction [9] is also a method used to enhance the contrast and brightness by simultaneously expanding dark regions and compressing bright ones. However, the main drawback of this method is that each pixel in the image is treated individually, without the dependence of their neighborhoods, thus providing inconsistent results with the real scenes. Image equalization technology is used for adjustments to resolve the aforementioned problems. Jenifer et al. [10] proposed a fuzzy cropping contrast-limited adaptive HE (CLAHE) algorithm to enhance the local contrast of the image and maintain image brightness. Singh [11] introduced an HE-based image enhancement method with highly adaptive group intelligence optimization to improve the overall image enhancement effect and retain the inherent detail information. Khan [12] used wavelet transform to decompose the image into low- and high-frequency portions. The contrast of the low-frequency part is adjusted using CLAHE, and the resulting image is processed using fuzzy contrast enhancement technology to maintain the spectral information of the image. Fu et al. [13] utilized a dual-branch network to compensate for the global color distortion and local contrast reduction and designed a compressed HE to supplement the data. In addition, variational methods that use different regularization terms on the histogram have been proposed. For example, contextual and variational contrast enhancement [14] attempts to find histogram mapping to obtain large gray-level differences. The second category is based on the Retinex theory proposed by Land and McCann in 1971 [15]. The dominant assumption of Retinex theory is that the image can be decomposed into reflection and illumination. Retinex theory has been widely studied and applied in the past four decades. The classic single-scale Retinex (SSR) [16], multiscale Retinex (MSR) [17], and multiscale Retinex with color restoration (MSRCR) [18] approaches are continuously improved and extended to obtain additional image information. Similar to the difference-of-Gaussian function, which is widely used in natural vision science, SSR based on the center/surrounding Retinex treats the reflectance as the final enhanced result. MSR is considered to be the weighted sum of several different SSR outputs. MSR not only maintains image fidelity and compresses the dynamic range of the image but also achieves color enhancement and invariance. MSRCR adds a color restoration factor based on the MSR to solve the problem regarding channel color ratio and adjust the local image contrast enhancement, thereby resulting in the color distortion defect. NASA also uses the Retinex framework technology in processing related images [19,20]. Several new algorithms based on Retinex theory were proposed. Seonhee Park et al. [21] used the variational optimization-based Retinex algorithm to enhance the low-illumination image. Li et al. [22] used a recursive bilateral filter instead of the traditional Gaussian function as the brightness estimation function to achieve brightness estimation. In addition to the lighting effect, Jung et al. [23] also used light normalization filtering to realize human eye detection in a wide range of lighting conditions. Fu et al. [24] proposed a weighted variational model called simultaneous reflectance and illumination estimation, which simultaneously estimates the illumination and reflection components. The last category is the method based on the dehazing model. Inspired by the dark channel method on dehazing, [25] identified the inverted low-illumination image similar to a haze image. They attempted to remove the inverted low-illumination image of haze by using the method proposed in [26] and then inverted this image again to obtain the final result. Xiao and Gan [27] used a guided joint bilateral filter to refine the initial atmospheric scattered light to generate a new atmospheric veil. They also utilized an atmospheric attenuation model to restore the scene brightness. Salazar et al. [28] proposed an efficient dehazing algorithm based on morphological operations and Gaussian filters. The abovementioned traditional algorithms provide important theoretical support to the research on low-illumination image enhancement and improve the visual quality to a certain extent.

The application of deep learning in image classification, recognition, and tracking has yielded positive results [29,30,31,32]. Deep learning builds network models by imitating the neural network of the human brain and using efficient learning strategies to obtain results through multilevel analyses and calculations. Literature [33] used convolutional neural networks (CNNs) to extract features at different levels and enhanced them through multiple subnets, outputting the images through multibranch fusion. Chao et al. used a four-layer convolutional network AR-CNN to demonstrate that a deep model can be effectively trained with the features learned in a shallow network [34]. Literature [35] presented a feed-forward fully convolutional residual network model trained using a generative adversarial framework (GAN). The results confirm the feasibility of deep learning methods. Several image enhancement algorithms related to deep learning were also proposed and rapidly developed in the past few years. Huang et al. [36] proposed a novel frame-wise filtering method based on CNNs. A novel multiframe CNN, in which the non-peak quality frames (PQFs) and the two nearest PQFs are the input, is designed to improve the image quality [37]. Zhang et al. proposed recursive residual CNN (RRCNN)-based in-loop filtering to further improve the quality of reconstructed intra frames while reducing the bitrates [38]. Chen et al. [39] proposed a low-illumination image processing pipeline based on the end-to-end training of a fully convolutional network, which can jointly process noise and color distortion. However, this pipeline is specific to RAW format data; such a condition limits its application in scenarios. Shen et al. argued that MRS is equivalent to a feedforward CNN with different Gaussian convolution kernels. They built a CNN called MSR-net [40] to learn the end-to-end mapping between dark and bright images. Wei et al. designed a deep network called Retinex-Net, which combines image decomposition and light mapping [41].

The above algorithms, whether based on traditional or deep learning, achieved excellent results. However, flexible ways for processing low-illumination images in spatial environments with sophisticated lighting are necessary. This study is based on the enhancement of the deep convolutional GAN (DCGAN) in the CIELAB color space to simulate the observed effect of the human eye. Overall, the contribution of this study is summarized in the following three aspects: (1) A deep neural network-based shimmering image enhancement method, which improves objective and subjective image quality, is proposed; (2) The use of CIELAB color space, which is consistent with the formation mechanism of human perception of color, enables the recovery of the entire image color to some extent; (3) The proposed method obtains bright and natural results, sharp textures, and rich details. Moreover, quantitative and qualitative evaluations demonstrate that the proposed method largely outperforms other methods.

The remainder of this paper is organized as follows. Section 2 introduces the related work of the algorithms used in this study, which mainly includes the GAN, DCGAN, and Wasserstein GAN (WGAN). Section 3 explains the network model proposed in this paper based on the WGAN loss function, and the loss function of the proposed network is improved to address the unstable GAN training. Section 4 introduces different low-illumination image processing algorithms and facilitates their comparison, and the results of low-illumination image processing under three different situations (general, special, and actual images) are analyzed. Section 5 provides several conclusions drawn from this research.

## 2. Related Work

### 2.1. CIELAB Color Model

Color information is affected by changes in illumination, which often influences subsequent robotic visual recognition. The commonly used RGB color space contains almost basic colors that human vision can perceive. However, despite the strong correlation between the components and the narrow color range, including only three channels of red (R), green (G), and blue (B), the ratio of the three components can easily change, complicating the processing of RGB color images; the color of the image also changes with the components [42]. The L component in CIELAB represents luminance perception, while the A and B components constitute prediction. CIELAB has a wide range of colors, from red to dark green for the “A” channel and from blue to yellow for the “B” channel, and is light-independent. The lightness and color information of CIELAB are separable and can be simply adjusted to achieve effects that can only be realized by complex steps in other color spaces [43]. Therefore, replacing the RGB color space with the CIELAB color space can enhance the accuracy of removing the environmental lighting information and retaining the essential characteristics of the target [44].

### 2.2. GAN, DCGAN, and WGAN

**Figure 1:**
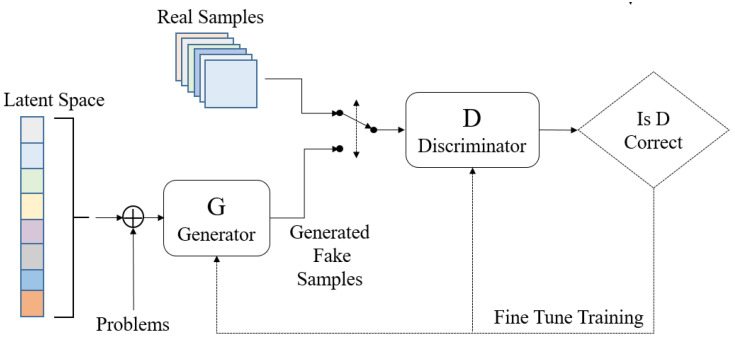
Flow chart of GAN.

GAN, an exclusive deep learning network based on the Nash balance in game theory, is a new network framework proposed by Goodfellow in 2014 [45]. This network comprises a generation network *G* and a discriminant network *D*, as shown in Figure 1. The function of G is to generate a series of realistic sample *G*(z) according to the random problem variable z to “deceive” *D*, and *D* can master the identification method of the sample by learning true sample x and *G*(z) generated by *G*. *D* and *G* are promoted synchronously in the mutual game process until the sample generated by *G* is realistic and *D* loses its function, thus failing to distinguish between true and false.

In the training process of *D* and *G*, one party is fixed, and the other party updates the weight. In this process, both parties attempt to optimize their networks to form a game confrontation and finally achieve a dynamic balance. The above process can be expressed as the maximum and minimization problem:

(1) $$\underset{G}{\min}\underset{D}{\max}V(D,G)=E_{x\sim P_{r}}[\log D(x)]+E_{z\sim P_{z}(z)}[\log(1-D(G(z)))]$$

In the above game problem, the training network for discriminant network *D* realizes the maximum probability of distinguishing the training sample label and maximizes logD(x), while that for generation network *G* results in log(1−D(G(z))) minimum and maximizes the loss of *D*. The game has the following objectives: fix one party during the training, update another network parameter, perform alternate iterations, maximize the disadvantages of each party, and finally enable the G to estimate the sample distribution of the real data.

The meanings of mathematical symbols in Equation (1) are shown in Table 1.

**Figure 2:**
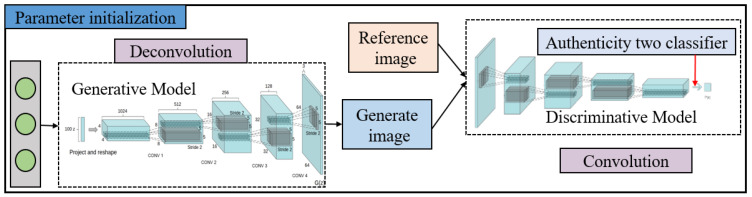
Network structure of DCGAN.

The principle of DCGAN is the same as that of GAN, and its process is similar to that of CNN. However, DCGAN replaces *G* and *D* with two CNNs. The model structure diagram is shown in Figure 2. The generation network receives a random input z and generates an image *G*(z) through upsampling, which mainly requires a deconvolution algorithm. The generation network is then converted into a 4 × 4 × 1024 feature map through full connection, and an image with a size of 64 × 64 × 3 is generated using multiple deconvolution layers. The input of the discriminant network is a picture. After the downsampling convolution, the image is connected to the fully connected layer for processing and then sent to the sigmoid function. The output is a true or false probability. DCGAN architecture does not fundamentally solve the instability of GAN training. Therefore, the training processes of the two networks should be carefully balanced.

WGAN discovered that Jensen–Shannon divergence caused GAN training instability and introduced a new distribution distance measurement method, namely, Wassertenin distance [46], which is also called Earth-Mover (EM) distance. This method represents the minimum cost of transforming from one distribution to another and can be obtained as follows:

(2) $$W(P_{r},P_{z})\approx \underset{\gamma \in \prod \left(P_{r},P_{g}\right)}{\inf}E_{(x,y)\sim \gamma }[\left\|x-y\right\|]$$

For each possible joint distribution γ, a sample x and y can be sampled from (x,y)~γ, and the distance ‖x−y‖ of the pair of samples can be calculated. Therefore, the expected value $E_{(x,y)\sim \gamma }[\left\|x-y\right\|]$ of the distance between the sample under the joint distribution γ can be calculated. In all possible joint distributions, the lower bound $\underset{\gamma \in \prod \left(P_{r},P_{g}\right)}{\inf}E_{(x,y)\sim \gamma }[\left\|x-y\right\|]$ of this expected value channel is the Wasserstein distance. A pair of samples are generally randomly taken from the generated and the real samples, and the mean value of the difference between these samples is calculated. The smallest mean value is the Wasserstein distance.

The definitions of mathematical symbols in Equation (2) are shown in Table 2.

## 3. Methodology

### 3.1. Low-Illumination Image Enhancement Algorithm Model

**Figure 3:**
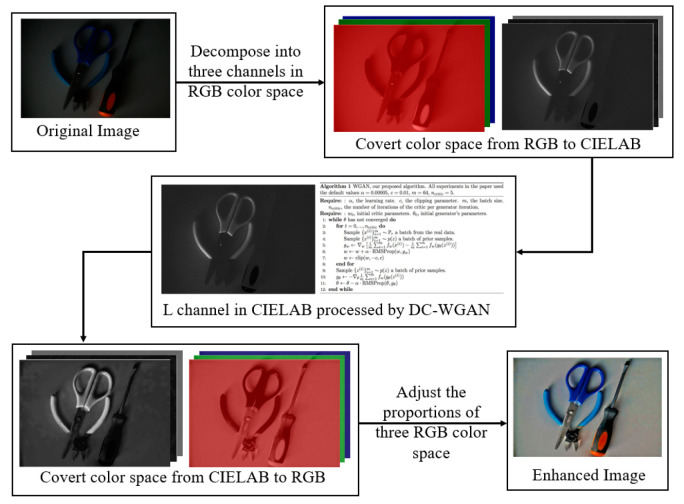
Flow chart of the proposed algorithm.

Low-illuminance images are enhanced using the advantages of color model transformation and combined DCGAN and WGAN (DC-WGAN) to address the weak effect of the current low-illumination image enhancement algorithm on the lighting conditions of spatially complex environments. The images are first transformed from the RGB space to the LAB color space. A and B are maintained, and the brightness component L is processed using DC-WGAN. The transformed image is then transferred back to the RGB space to obtain the final enhanced image. Compared with DCGAN, the DC-WGAN effectively solves the instability problem of GAN training. The DC-WGAN uses low-illuminance images as input to transform unsupervised image generation into supervised image enhancement (equivalent to the vector constraints added by the DCGAN encoder) to allow the network to output enhanced images under specific conditions. The proposed algorithm flow is illustrated in Figure 3.

### 3.2. DC-WGAN Structure

**Figure 4:**
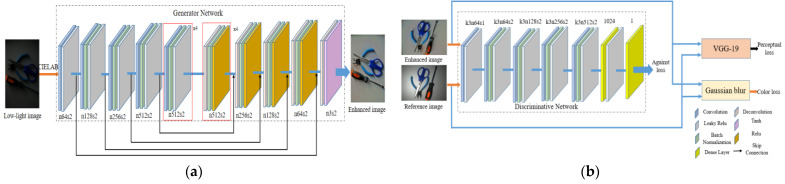
Adversarial generation network model for low-light images: (**a**) generator network; (**b**) discriminative network.

The DC-WGAN is an improved CNN with encoding and decoding functions. WGAN with a measurement network function is added as a discriminative model. The GAN formation follows the confrontation learning idea. The generation network is responsible for producing the enhanced image, whereas the discriminant network distinguishes the real image from the image produced by the generation network. Both networks play a game with each other. Figure 4 depicts the network structure diagram, which includes the generation and discriminant networks and the loss function.

#### 3.2.1. Generation Network

The DC-WGAN (Figure 4a) maintains the high-level design of DCGAN, which is divided into upsampling and downsampling. Downsampling goes through eight ordinary convolutional layers for eight downsampling operations to extract rich features from the input image. Sparse coding is then performed to reduce the spatial dimension of the image features. Downsampling uses eight deconvolutional layers to perform the image upsampling to restore image details. Except for the convolutional layer directly connected to the image, all layers adopt batch normalization operations. Ordinary convolutional layers use the LeakyRelu activation function to reduce the number of iterations and avoid the disappearance of the gradient. The deconvolution layer uses the Relu activation function to simplify the calculation. The filling method adopts the same filling, that is, the layer is filled with 0 after the convolution operation until the size is consistent with the original. The normal convolutional and deconvolutional layers with the same output size are jumped to prevent the loss of features. For the convolution layer directly connected to the image, the convolution kernel adopts a size of 1 × 1 for channel adjustment, and the corresponding patch size on the image is 1 × 1. For other convolutional layers, the convolution kernel adopts the size of 3 × 3, and the patch size corresponding to the previous feature map is 3 × 3. The convolution kernel obtains information for each patch of the image through the convolution operation with a step size of 2. n512s2 represents a convolution with a step size of 2, the number of output channels is 512, and ×4 represents a convolution that is repeated four times. No further increase in the number of channels is observed due to the sufficient information of 512 channels. Finally, deconvolution and Tanh function are added to the last layer of the generation network to improve the stability of network training and restore high-quality image features to obtain enhanced images.

#### 3.2.2. Discriminant Network

As a two-classification network, the discriminant network of DCGAN (Figure 4b) comprises a sigmoid activation function to obtain the probability of the category. The idea of WGAN is adopted in this model. The discriminator in WGAN is used as the EM distance measurement network to classify DCGAN. The problem is transformed into a regression problem. The objective is to measure the EM distance, that is, to determine the Wasserstein distance of the two distributions under the real number space. The sigmoid activation function of the last layer is unnecessary and is therefore removed. The DC-WGAN discriminant network is shown in Figure 4. The first layer of the convolutional layer extracts the underlying feature information, and the convolution step is 1. This layer comprises four similar structural blocks, each having convolutional and batch normalization layers. The leaky rectified linear unit (ReLU) activation module is downsampled with a step size of 2 to increase the visual information of the feature map during the extraction of high-dimensional features. Finally, the 2D feature map is converted into a 1D feature vector by using a fully connected layer with a dimension of 1024.

#### 3.2.3. Loss Function

The adversarial loss function in the DC-WGAN algorithm proposed in this paper is based on the WGAN algorithm. A brief description of the WGAN loss function derivation process is presented as follows to understand the DC-WGAN adversarial loss function.

Traversing all joint distributions γ to calculate the expected $E_{(x,y)\sim \gamma }[\left\|x-y\right\|]$ of ‖x−y‖ is impossible based on the previously mentioned characteristics of WGAN. The author of the WGAN based on the Kantorovich–Rubinstein duality used Lipschitz continuity to solve the Wasserstein distance and redefine it as follows [36]:

(3) $$W(P_{r},P_{g})\approx \frac{1}{K}\underset{{\left\|f\right\|}_{L}\leq K}{\sup}E_{x\sim P_{r}}[f(x)]-E_{x\sim P_{g}}[f(x)]$$

Equation (3) denotes that when sample x is sampled from the real data distribution $P_{r}$, the expected value after the f(x)-transformation should be as large as possible to maximize $E_{x\sim P_{r}}[f(x)]-E_{x\sim P_{g}}[f(x)]$. Similarly, when the sample x is takenfrom the generated sample distribution $P_{g}$, the expected value after the f(x)-transformation should be as small as possible. However, $E_{x\sim P_{r}}[f(x)]$ and $E_{x\sim P_{g}}[f(x)]$ cannot be respectively endlessly large and small simultaneously. Moreover, the loss function will increase, and convergence will never be achieved. Therefore, a continuous Lipschitz limit on the function f(x) is needed. A K-order Lipschitz continuity is defined as follows:

(4) $$\left|f(x_{1})-f(x_{2})\right|\leq K\cdot \left|x_{1}-x_{2}\right|$$

Lipschitz continuously describes that the derivative of a function at any point does not exceed the constant *K*, indicating the smoothness of the function and the absence of sudden gradients. This restriction allows the loss function convergence, and *K* = 1 is generally chosen.

The function f(x) uses the discriminant network to approximate the fitting due to the strong capability of the discriminant network to fit the function. The parameter of this function is assumed to be ω. When $f_{\omega }(x)$ satisfies the first-order Lipschitz constraint, Equation (3) is simplified as follows:

(5) $$W(P_{r},P_{g})\approx \underset{{\left\|f_{\omega }\right\|}_{L}\leq 1}{\sup}E_{x\sim P_{r}}[f_{\omega }(x)]-E_{x\sim P_{g}}[f_{\omega }(x)]$$

Therefore, the problem of solving $W(P_{r},P_{g})$ can be transformed into:

(6) $$\underset{\omega }{\max}E_{x\sim P_{r}}[f_{\omega }(x)]-E_{x\sim P_{g}}[f_{\omega }(x)]$$

Equation (6) is the optimization goal of discriminator *D*. When training the discriminator, WGAN considers the loss function to be a de-maximization that satisfies the Lipschitz continuous constraint:

(7) $$L=E_{x\sim P_{r}}[f_{\omega }(x)]-E_{x\sim P_{g}}[f_{\omega }(x)]$$

The definitions of mathematical symbols from Equations (3)–(7) are shown in Table 3.

Low-illumination image enhancement is inevitably accompanied by other problems, mainly noise and blurred details. The global residuals in the GAN can effectively deal with the noise problem but does not improve the blurring of the generated image. Perceptual and color losses are also introduced to further improve the quality of the enhanced image. Therefore, the loss function of DC-WGAN is derived from the weighted value of perceptual ($L_{p}$), adversarial ($L_{adv}$), and color losses ($L_{c}$). Weights are chosen to balance the relationship between each loss, and the most direct way to adjust the parameter values of weights is generally used to fit the neural network model and obtain an improved model structure.

The perceptual loss [47] is used to help restore the image content. This perceptual loss is based on the difference in feature mapping between the enhanced and reference images. The perceptual loss is calculated on the basis of the feature map generated by the ReLU-5-4 layer of the pre-trained VGG-19 network [48]. Perceptual loss is defined as follows:

(8) $$L_{p}=\frac{1}{n}\sum_{i=1}^{n}{\left\|\phi_{j}(I_{enh}{}_{i})-\phi_{j}(I_{refi})\right\|}_{2}^{2}$$

The adversarial loss function from WGAN-GP is used to enhance the convergence in the training process, and a gradient penalty (GP) [33] is added to each sample independently in WGAN to allow the discriminant network to satisfy the first-order Lipschitz function constraint. This loss function is defined as:

(9) $$L_{adv}=E_{x_{r}\sim P_{r}}D(x_{r})-E_{x_{f}\sim P_{g}}D(x_{f})-\lambda E_{x_{m}\sim P_{m}}[{\left({\left\|\nabla_{x_{m}}D(x_{m})\right\|}_{2}-1\right)}^{2}]$$

The gradient will stabilize around 1 after the discriminant network is fully trained. The gradient can be stabilized by adding GP, and the loss during training can be reduced.

The application of a Gaussian blur and the computation of Euclidean distance between the obtained representations are proposed to measure the color difference between the enhanced and reference images. In the context of CNNs, this approach is equivalent to using one additional convolutional layer with a fixed Gaussian kernel followed by the mean squared error (MSE) function. Color loss [49] can be written as:

(10) $$L_{c}=\frac{1}{n}\sum_{i=1}^{n}{\left\|I_{enhbi}-I_{refbi}\right\|}_{2}^{2}$$

The overall loss function of the model is the weighted average of all losses:

(11) $$L_{loss}=\lambda_{p}L_{p}+\lambda_{adv}L_{adv}+\lambda_{c}L_{c}$$

where $\lambda_{p}$, $\lambda_{adv}$,and $\lambda_{c}$ are the weights of each loss function.

The definitions of mathematical symbols from Equations (8)–(11) are shown in Table 4.

## 4. Experiments and Results

The proposed method is evaluated and compared with the existing methods. The public codes of the existing approaches are used for comparison. Three experiments are conducted. First, the proposed algorithm for the general dataset is compared with several existing representative algorithms considering low-illumination image strength. Second, the dataset is established, and another group of low-illumination image enhancement comparisons is performed. Last, the low-illumination graphics of the real space environment are used to illustrate the experimental results.

### 4.1. Used Data

Deep learning-based low-illumination image enhancement is still inits infancy because deep learning-related methods require the production of a large number of training samples. The current low-illumination processing methods require the acquisition of normal and low-illumination images in the same scene because the input of the neural network differs from person to person in normal light recognition. Thus, this requirement severely restricts the research on deep learning in the field of image enhancement. This study proposes an easy-to-operate and non-time-consuming training sample generation method.

Distortions were eliminated, images that were considerably large, small, or inappropriate were excluded, and 1200 improved source images were obtained from the VOC [50] dataset. Images that are substantially large result in a large computation of the CNN, which cannot be satisfied by the existing hardware. By contrast, considerably small multiple neural network convolutions will affect the accuracy. Therefore, the input image size must not exceed 600 × 600 at the maximum and lower than 300 × 300 at the minimum. The Photoshop method is then used to process each image to obtain the vision of high-quality images with improved results (reference images). The brightness of each image is then reduced, and random parameter gamma is used to generate 10 low-illumination images. Therefore, 12,000 pairs of high-quality/low-illumination image datasets are obtained, and 10,000 sheets are selected in the dataset. The public low-illumination image dataset SID [39] and LOL [41] dataset real-world low-illumination images are chosen to evaluate the proposed method objectively and fairly. A total of 16,980 images are selected from the three datasets to synthesize a dataset with enhanced generalization capability for network training and testing. Roughly 95% of the data constitute the training set, and the rest accounts for the test set. The robot should be trained to recognize the target tools because space robots will replace astronauts in the maintenance work inside and outside the cabin. Approximately 6200 images of target tools are collected under simulated space lighting conditions to build a dedicated dataset, in which 90% form the training set, and the rest comprises the test set. Directly inputting the model for training is inconvenient due to the large size of the image dataset. The image size resolution in the dataset is adjusted to 600 × 400, and some images are randomly flipped up and down and left and right to increase the diversity of the training images.

### 4.2. Experimental Conditions

The computer graphics processing unit is an NVIDIA GeForce RTX 2080Ti, while the central processing unit is Intel Core i9-10900F. The TensorFlow deep learning framework is used for training. The generative and discriminative models are optimized using an Root Mean Squareprop (RMSProp) optimizer. The learning rate in the first 20 cycles is set to 10^−4^ and then attenuated to 10^−5^. The generative and discriminant networks are trained alternately.

**Figure 5:**
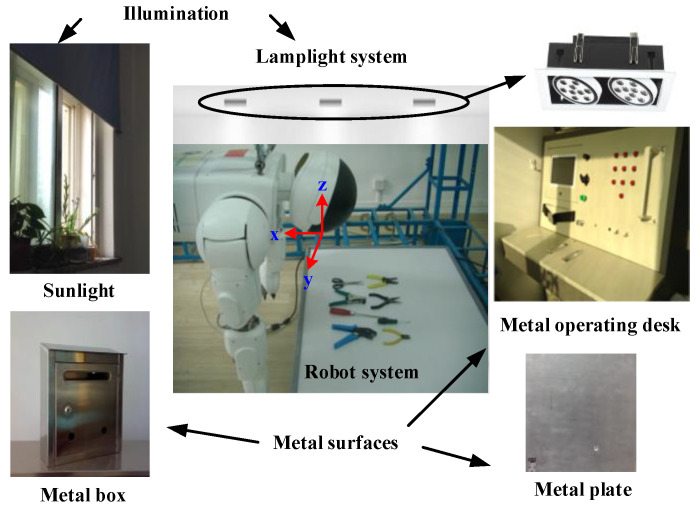
Lighting simulation system.

A lighting simulation system is established on the ground in cooperation with Beijing Insitute of Technology (Figure 5) to simulate the lighting environment where the intelligent robot system is located. The system mainly includes natural and LED lights, and the reflection of the metal surface is also considered in the ground laboratory to increase the closeness of the collected images to the real state. The LED lighting system is divided into three levels: strong, medium, and weak. Meanwhile, natural light was divided into daylight and no daylight by controlling the curtain switch. Three time periods (9:00 AM, 4:00 PM, and 8:00 PM every day in mid-July) were selected for image acquisition. A total of 20,000 images were collected by adjusting the LED lighting system to correspond to the natural light at different times, and 6200 images meeting the requirements were selected as the dedicated dataset.

### 4.3. Experimental Results and Analysis

**Figure 6:**
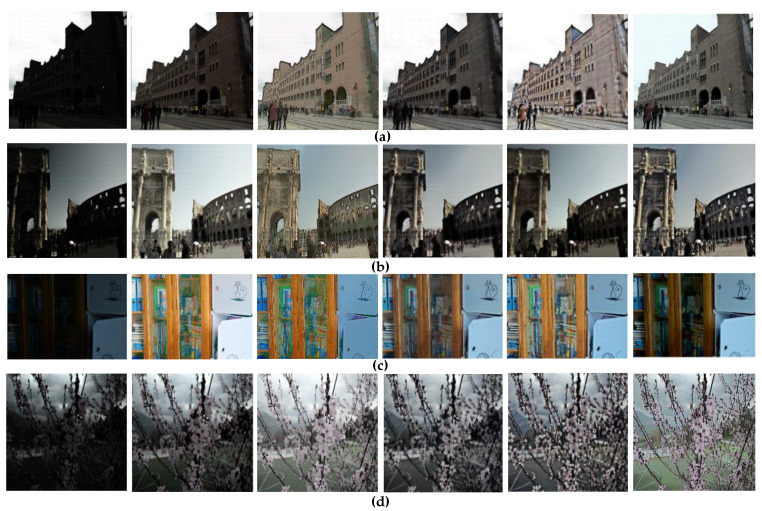
Low-illumination MSRCR RetinexNet KinD MBLLEN Ours. Comparison of the processing effects on the general dataset. the leftmost column is the low-illumination image, and the other four columns are the results of the low-illumination image processed by MSRCR, RetinexNet, KinD, MBLLEN and our proposed algorithms.

**Figure 7:**
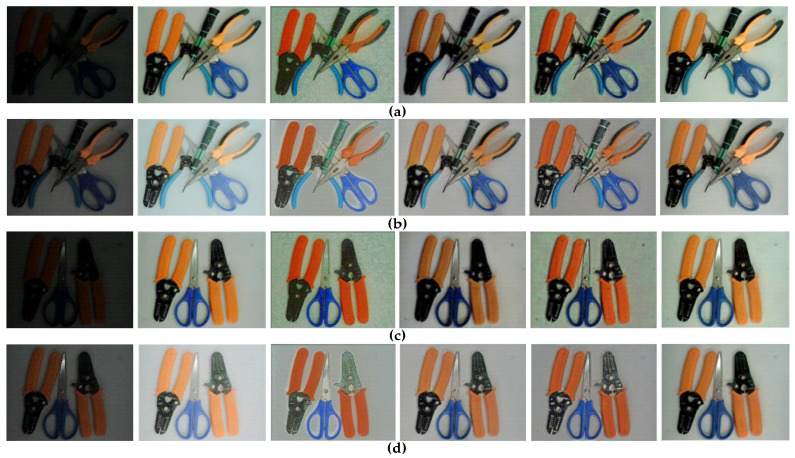
Low-illumination MSRCR RetinexNet KinD MBLLEN Ours.Comparison of the processing effects on the special datasets: (**a**,**b**) pictures of the same tool under different lighting conditions and (**c**,**d**) pictures of the same tool under different lighting conditions.

The performance of the proposed algorithm is compared with those of the existing low-illumination image enhancement methods. Only the algorithms with satisfactory results, namely MSRCR, Retinex-Net, KinD [51], and MBLLEN algorithms [52], are selected due to GPU memory limitations. The proposed algorithm is applied to the existing general dataset, and its performance is compared with the other algorithms on the dedicated dataset. Displaying all the images is impossible due to the excessive number of images in the test set. Therefore, only one image under four scenes (Select 20 images under each type of scene) is selected as a representative, and shown in Figure 6 and Figure 7.

Regardless of the type of dataset (general or unique dataset), the subjective evaluation (i.e., inspection through the human eye) indicates that the proposed algorithm performs better than the traditional algorithms considering overall clarity, color reproduction, and image detail information. However, establishing a convincing assessment based on subjective evaluation is difficult. Therefore, an objective evaluation of the brightness, contrast, and information entropy is conducted [53]. The average results for the four types of scenarios are presented in Table 5 and Table 6.

The data in Table 5 must be compared horizontally, corresponding to Figure 6. This comparison aims to change the same type of image after processing by different algorithms, and the values of lightness, contrast, and information entropy will also change. The analysis of these values helps determine the best algorithm based on the same image processing result. Therefore, the algorithm must be changed under the condition of maintaining the others to ensure fairness and truthfulness of the comparison. Table 5 shows that the brightness, contrast, and information entropy of the general original image are the smallest. This result indicates that extracting the target feature information is difficult due to the interference of the low-illuminance environment. The information value becomes the largest after using the DC-WGAN algorithm, exhibiting an increase of nearly 20% compared with the original low-illuminance image. The contrast and brightness are also improved, and the target feature information is enhanced. The best value of image brightness is 128 in most cases. After applying the DC-WGAN algorithm, the brightness values of the images presented in Figure 6a–d increase from 32.9625, 49.6086, 24.8556, and 40.9445 to 120.9182, 131.3102, 128.1027, and 127.0935, respectively. These values are the closest to 128 among the values obtained using the five compared algorithms.

Table 6 shows that the original low-illuminance images with the same lighting conditions have almost the same brightness, contrast, and information entropy and large differences among various lighting conditions. The brightness values of the resulting images nearly reach 128 after the application of the proposed algorithm, and the corresponding contrast values are the largest among the obtained values. Similarly, the information entropy of the images shown in Figure 7a–d increases by roughly 45%, 13%, 44%, and 13.6% compared with the original versions. Compared with the four traditional algorithms, the DC-WGAN algorithm can restore additional details and demonstrates the best enhancement performance.

Overall, Table 5 and Table 6 are objective evaluations of the lightness, contrast, and information entropy of the processed images. However, the only difference is that the processing objects are different. The data analysis in the table can roughly conclude that the proposed algorithm is effective for these types of images on general and dedicated datasets.

**Figure 8:**
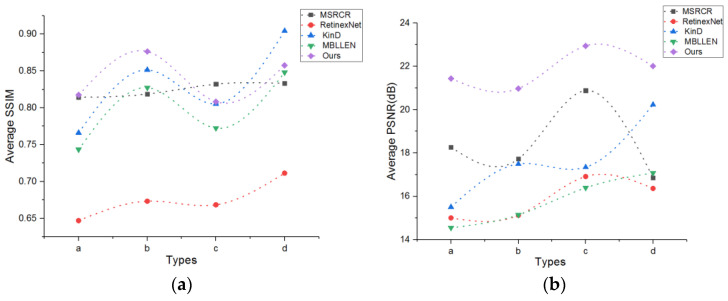
Objective performance evaluation of the different algorithms in special images considering; (**a**,**b**) are the average SSIM, and average PSNR for the four types of scenes. The original discrete points are fitted into a curve to provide an enhanced viewing effect.

For dedicated dataset, an objective assessment considering peak signal-to-noise ratio (PSNR) and structural similarity index measure (SSIM) is performed to obtain a fair performance evaluation of the different methods [54]. The processed image is compared with the reference image. High PSNR values result in good image quality. SSIM is an index that measures the similarity of two images. The values range between 0 and 1. When the SSIM is 0, the two images are not correlated; by contrast, when the SSIM is 1, the two images are considered the same. Figure 8 shows that although other algorithms have slightly higher PSNR and SSIM values than the DC-WGAN algorithm, the overall effect of the latter is slightly better than that of the former.

The aforementioned dedicated dataset brightness, contrast, information entropy, and the objective evaluation of PSNR and SSIM indicate that the DC-WGAN algorithm has better performance than other compared algorithms, followed by MSRCR and MBLEEN algorithms.

A time-consuming algorithm will be discarded over time despite its good performance. Therefore, the processing time should also be assessed to achieve a comprehensive evaluation and ensure that an algorithm not only has improved performance but also speed. Ten classes of images (20 images per class) are selected from a dedicated dataset. The size of low-illuminance images is adjusted to the three-pixel sizes (600 × 400, 500 × 400, and 400 × 300), and different pixel sizes under the same image conditions are used to compare the average processing time of different algorithms. The selected top three are objectively evaluated in the dedicated dataset for the comparison of processing times. The results are summarized in Table 7.

The results reveal that although the size of the image will affect the image processing time, the proposed algorithm is faster than the MSRCR and MBLLEN algorithms in all image sizes. This result not only reflects the advantages of the former but also promotes the development of deep learning in preprocessing.

**Figure 9:**
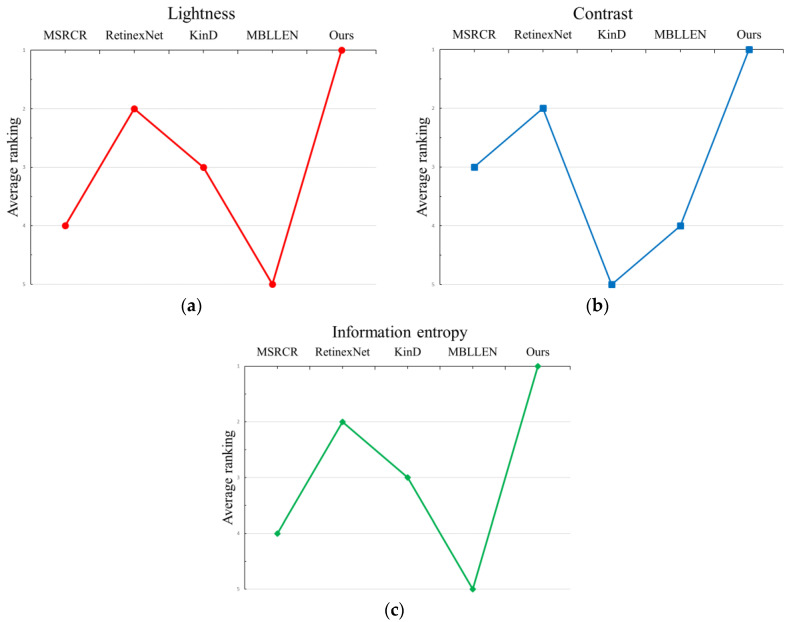
Average ranking of the two test sets, namely general and dedicated, on lightness, contrast, and information entropy: (**a**) for lightness ranking; (**b**) for contrast ranking and (**c**) for information entropy ranking.

The MSRCR, RetinexNet, KinD, MBLLEN, and DC-WGAN algorithms are run on both test sets to verify the combined performance of the proposed DC-WGAN algorithm compared with other existing algorithms in general and dedicated test sets. The brightness, contrast, and information entropy obtained by different algorithms on each image are calculated, and all algorithms are ranked according to the obtained results. Figure 9 shows that the DC-WGAN algorithm achieved good results in all three evaluation metrics, in which the average performance is better than other algorithms in lightness and information entropy. The proposed algorithm is also slightly inferior to MSRCR. However, the MSRCR algorithm performed relatively poorly in other metrics. Overall, the DC-WGAN algorithm shows good generality and can cope with most low-illumination scenes. On the previously dedicated dataset, the DC-WGAN algorithm is again verified to be better than other algorithms on the dedicated dataset by the average SSIM, PSNR, and average processing time for several types of images.

**Figure 10:**
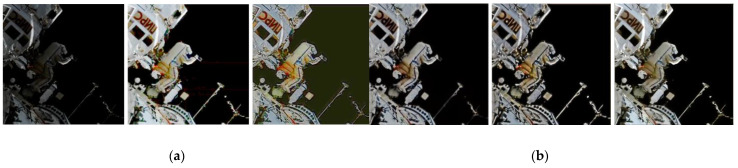
Different algorithms in low-illumination environments that can be used by astronauts for extravehicular space image enhancement. (**a**) Low-illumination MSRCR RetinexNet; (**b**) KinD MBLLEN Ours.

The superiority of the proposed algorithm over other approaches in general or special datasets is confirmed. The dataset collected through simulation is different from the real space station environment. Thus, the existing aerospace video data can be used by aerospace operators that work in a low-illuminance and high-radiation environment to perform centralized algorithm processing (Figure 10). Through the application in the actual space environment, Figure 10 shows that the pictures processed by the DC-WGAN algorithm will not produce additional noise, blurring, and other problems during the processing. The color areas in the picture are also enhanced, which is consistent with the characteristics of the human eye. Thus, the proposed algorithm is better than other compared algorithms.

## 5. Discussion and Conclusions

This study proposes the DC-WGAN algorithm to address the difficulty of visual positioning caused by low illumination light during robot operations in space. The feasibility of the proposed algorithm is verified by the research on low-illuminance image algorithms and experiments under general, special, and actual conditions. Two main conclusions are obtained. First, the enhanced form based on the CIELAB color space and DC-WGAN brightness component is conducive for enhancing the color to a level that is close to the characteristics of the human eye. Second, double-layer networks can obtain many image features. The rich features extracted by the different layers of the network can be appropriately mapped to the denoised image, and the difference between the obtained and reference images is small. Moreover, different low-illumination image processing algorithms are compared and investigated, and the results of low- illumination image processing under three different situations (general, special, and actual images) are analyzed to ensure that the proposed algorithm is comprehensive and effective.

Overall, the proposed algorithm in this paper achieved good enhancement effects, reducing the processing time of each frame image and enriching image detail information in low-illumination environments. The results fully prove the feasibility and theoretical significance of the proposed scheme engineering. Moreover, the results provide research considering target identification and on-orbit servicing in the space environment.

## Figures and Tables

**Table 1:** Parameters in the mechanical model.

Symbol	Comment	Symbol	Comment
x	True sample	D(x)	The output of the true sample x in the discriminator network
z	Random question	G(z)	The output of the random problem variable z in the generator network
$P_{r}$	Distribution of the true sample	$P_{z}(z)$	Some prior distribution (generally Gaussian distribution)
$P_{z}$	Distribution of generated sample by generator network	$E_{x\sim P_{r}}$	Meet the expectation under the real data distribution
E	Expectation(or Mean)	$E_{z\sim P_{z}(z)}$	Meet the expectation under the data distribution generated by the generator network

**Table 2:** Parameters in the mechanical model.

Symbol	Comment	Symbol	Comment
x	True sample	(x,y)~γ	True sample x and generated sample y that obey the joint distribution
y	Generated sample	$E_{(x,y)\sim \gamma }$	The expectation of true sample x and generated sample y that obeys the joint distribution.
$P_{r}$	Distribution of the actual sample	$\prod (P_{r},P_{g})$	The set of all possible joint distributions of the true sample distribution $P_{r}$ and the generated sample distribution $P_{g}$
$P_{g}$	Distribution of generated sample	inf{⋅}	The lower bound of the set
γ	Joint distribution	‖x−y‖	The distance between the true x sample and the generated sample y

**Table 3:** Parameters in the mechanical model.

Symbol	Comment	Symbol	Comment
sup{⋅}	Supremum of the set	ω	Discriminant network parameter
f(x)	Discriminant network function	$f_{\omega }(x)$	Parameterized discriminant network function
K	Lipschitz constant of the function f(x)	${\left\|f\right\|}_{L}$	Lipschitz norm

**Table 4:** Parameters in the mechanical model.

Symbol	Comment	Symbol	Comment
n	Number of input images per iteration	$x_{r}$,$x_{f}$	samples r,f
ϕ	Pre-trained convolutional neural network	$x_{m}$	The linear difference between $x_{r}$ and $x_{f}$
$\phi_{j}$	Features extracted from the j-th convolutional layer in the VGG19 network	$P_{m}$	The uniform distribution of the entire space
I	Input image	λ	The coefficient, which is generally taken as 10
$I_{enhi}$	The i-th input image after model enhancement.	${\left\|\nabla_{x_{m}}D(x_{m})\right\|}_{2}$	The gradient of discriminant networks
$I_{refi}$	The i-th reference image in the training set	$E_{x_{m}\sim P_{m}}[{\left({\left\|\nabla_{x_{m}}D(x_{m})\right\|}_{2}-1\right)}^{2}]$	Gradient penalty
$\phi_{j}(I_{refi})$	Feature map of the j-th convolutional output of the i-th reference image	$I_{enhbi}$	Enhanced image is Gaussian blurred
$\phi_{j}(I_{enhi})$	Feature map of the j-th convolutional output of the i-th enhanced image	$I_{refbi}$	Reference image is Gaussian blurred

**Table 5:** Performance comparisons of the algorithms in general pictures under low illumination.

Universal	Low-Illumination	MSRCR	RetinexNet	KinD	MBLLEN	Ours
Lightness (a)	32.9625	172.9182	145.7661	79.1987	61.3455	**120.9182**
Lightness (b)	49.6086	159.4251	146.3468	93.1317	90.5047	**131.3102**
Lightness (c)	34.8556	148.5200	144.4201	105.5388	76.8041	**128.1027**
Lightness (d)	40.9445	150.1411	135.0935	91.4465	79.1938	**127.0935**
Contrast (a)	41.2120	47.67887	40.3747	45.2993	**51.7349**	49.6785
Contrast (b)	54.0318	**73.9437**	53.1560	65.9201	70.1734	67.3353
Contrast (c)	24.7853	64.8938	48.1577	52.1608	64.0822	**69.1070**
Contrast (d)	49.4502	58.0430	50.1791	56.4303	**60.6560**	56.6245
Information entropy (a)	6.4982	7.7346	7.6844	7.5220	7.0540	**7.7489**
Information entropy (b)	6.3197	7.3137	7.4510	7.3927	7.4706	**7.5427**
Information entropy (c)	6.2310	7.8280	7.4886	7.5260	7.3417	7.5285
Information entropy (d)	6.4706	7.5948	7.6199	7.6546	7.5899	**7.7071**

**Table 6:** Performance comparisons of the algorithms for dedicated pictures under low illumination.

Universal	Low-Illumination	MSRCR	RetinexNet	KinD	MBLLEN	Ours
Lightness (a)	17.0823	122.3738	113.5185	106.5485	105.6938	**132.3883**
Lightness (b)	54.2985	162.7967	137.6324	**128.2479**	119.4229	129.6730
Lightness (c)	18.5934	137.2348	117.7857	112.2623	110.7793	**131.0227**
Lightness (d)	55.3984	171.3505	137.9449	132.8697	121.9818	**126.9058**
Contrast (a)	12.4050	56.4434	51.8138	57.2679	52.4185	**61.9815**
Contrast (b)	25.5386	45.4280	42.2647	46.7885	41.2022	**55.3903**
Contrast (c)	12.2361	54.6097	51.3713	54.7486	51.0929	**60.3971**
Contrast (d)	25.6103	41.9631	39.5106	43.9869	39.5857	**55.0064**
Information entropy (a)	5.0223	6.8212	7.2580	7.0117	7.1515	**7.2996**
Information entropy (b)	6.2797	6.8337	6.8473	6.6038	6.5598	**7.1627**
Information entropy (c)	5.0042	6.7485	7.1906	6.7986	7.0823	**7.2070**
Information entropy (d)	6.2444	6.5725	6.5788	6.2113	6.3982	**7.0931**

**Table 7:** Comparison of the processing times of MSRCR, MBLLEN, and DC-WGAN algorithms for different picture sizes.

Time/s	600 × 400			500 × 400			400 × 300		
	MSRCR	MBLLEN	Ours	MSRCR	MBLLEN	Ours	MSRCR	MBLLEN	Ours
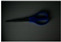	1.02911	0.10585	**0.06253**	1.40424	0.08967	**0.05675**	0.38865	0.05224	**0.03522**
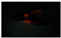	1.03613	0.10709	**0.06246**	0.42918	0.094667	**0.05336**	0.39429	0.05105	**0.03307**
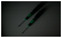	1.00008	0.10592	**0.06248**	0.43206	0.09056	**0.05362**	0.39170	0.05142	**0.03298**
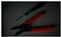	0.98677	0.10678	**0.06145**	0.43409	0.08674	**0.05331**	0.40789	0.05222	**0.03309**
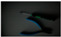	1.02807	0.10525	**0.06302**	0.42783	0.08849	**0.05352**	0.40448	0.05095	**0.03242**
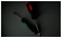	0.90992	0.10595	**0.06188**	1.04316	0.09288	**0.05362**	0.39798	0.05087	**0.03197**
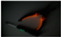	1.06087	0.10570	**0.06192**	0.90105	0.08830	**0.05503**	0.39812	0.05323	**0.03261**
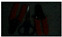	1.08931	0.10627	**0.06146**	0.976810	0.08991	**0.05347**	0.39924	0.05123	**0.03282**
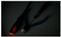	0.98742	0.10814	**0.06360**	0.429691	0.08821	**0.05310**	0.40571	0.05104	**0.03288**
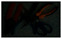	0.709238	0.106059	**0.06189**	0.43637	0.08830	**0.05315**	0.40176	0.05114	**0.03270**

## Data Availability

Data available in a publicly accessible repository.
